# Ultrasound Is Beneficial to Determine Lymphadenopathy in Oral Cancer Patients after Radiotherapy

**DOI:** 10.3390/diagnostics13142409

**Published:** 2023-07-19

**Authors:** Ping-Chia Cheng, Chih-Ming Chang, Li-Jen Liao, Chen-Hsi Hsieh, Pei-Wei Shueng, Po-Wen Cheng, Wu-Chia Lo

**Affiliations:** 1 Department of Otolaryngology Head and Neck Surgery, Far Eastern Memorial Hospital, New Taipei City 22060, Taiwan; i.cruising@gmail.com (P.-C.C.); b88401077@ntu.edu.tw (C.-M.C.); deniro@mail2000.com.tw (L.-J.L.); powenjapan@yahoo.com.tw (P.-W.C.); 2 Head and Neck Cancer Surveillance and Research Study Group, Far Eastern Memorial Hospital, New Taipei City 22060, Taiwan; chenciab@gmail.com (C.-H.H.); shuengsir@gmail.com (P.-W.S.); 3 Department of Communication Engineering, Asia Eastern University of Science and Technology, New Taipei City 22061, Taiwan; 4 Department of Biomedical Engineering, National Yang Ming Chiao Tung University, Taipei 11221, Taiwan; 5 Department of Electrical Engineering, Yuan Ze University, Taoyuan 32003, Taiwan; 6 Medical Engineering Office, Far Eastern Memorial Hospital, New Taipei City 22060, Taiwan; 7 Division of Radiation Oncology, Department of Radiology, Far Eastern Memorial Hospital, New Taipei City 22060, Taiwan; 8 Department of Medicine, School of Medicine, National Yang-Ming University, Taipei 11221, Taiwan; 9 Graduate Institute of Medicine, Yuan Ze University, Taoyuan 32003, Taiwan

**Keywords:** lymphadenopathy, head and neck ultrasound, fine needle aspiration, magnetic resonance imaging (MRI), computed tomography (CT)

## Abstract

The present study aimed to investigate whether the addition of ultrasound (US) +/− fine needle aspiration (FNA) to magnetic resonance imaging (MRI) or computed tomography (CT) improves the diagnostic accuracy in assessing neck lymphadenopathy in oral cancer patients after neck irradiation. We retrospectively reviewed oral cancer patients who had neck lymphadenopathy after radiotherapy (RT) or chemoradiation therapy (CRT) from February 2008 to November 2019. The following diagnostic modalities were assessed: (1) MRI/CT, (2) MRI/CT with a post-RT US predictive model, and (3) MRI/CT with US + FNA. The receiver operating characteristic (ROC) curves were used to assess the diagnostic performance. A total of 104 irradiation-treated oral cancer patients who subsequently had neck lymphadenopathy were recruited and analyzed. Finally, there were 68 (65%) malignant and 36 (35%) benign lymphadenopathies. In terms of the diagnostic performance, the area under the ROC curves (C-statistics) was 0.983, 0.920, and 0.828 for MRI/CT with US + FNA, MRI/CT with a post-RT US predictive model, and MRI/CT, respectively. The addition of US to MRI/CT to evaluate cervical lymphadenopathy could achieve a better diagnostic accuracy than MRI/CT alone in oral cancer patients after neck irradiation.

## 1. Introduction

In patients with radiotherapy (RT)-treated oral cancer, the early recognition of recurrent cervical lymphadenopathy is important in improving the success rates of salvage treatment and the final prognosis [1]. RT or chemoradiation therapy (CRT) is the standard treatment modality for advanced oral cancers and usually applied as adjuvant therapy after the operation. However, some earlier studies have indicated that prior RT or neck dissection can cause substantial alterations to the neck structure, such as tissue fibrosis or necrosis, which further lead to the difficulty in assessing an enlarged cervical LN [2,3,4]. The regional recurrence rate of oral cancers following treatment ranges from 11% to 51.1% [4,5,6], and malignant lymphadenopathy mostly occurs within 2 years [6]. Clinically, we often depend on various types of imaging studies, such as ultrasound (US), magnetic resonance imaging (MRI), and computed tomography (CT), to detect possible nodal recurrence after the treatment of oral cancer [7,8,9]. For patients with advanced head and neck cancers after complete treatment, the guidelines of the 2022 National Comprehensive Cancer Network suggest various imaging methods, including US, MRI, CT, and positron emission tomography (PET), each having their own advantages during the surveillance period [10]. Currently, the NCCN guidelines have recommended follow-up intervals for MRI, CT, and PET scans, whereas US is usually reserved as the assistant examination, and no follow-up interval is suggested for US. A US is a simple, fast, low-cost, and noninvasive tool that can depict superficial tissue very clearly without the risk of radiation exposure. Most importantly, when there are suspicions of recurrent cervical lymphadenopathy during the examination, US-guided needle aspiration can be executed for tissue sampling [11,12]. Nishimura et al. [13] showed that, for diagnosing recurrent cervical LN in head and neck cancer patients following CRT, the sensitivity and specificity was 88.2% and 66.1% for US, 52.9% and 74.2% for MRI/CT, and 71.4% and 95.6% for fine needle aspiration (FNA), respectively. For the surveillance of regional recurrence in 36 patients with treated oral cancers, Wierzbicka et al. [14] reported that the sensitivity and specificity was 79% and 94% for PET/CT and 83% and 83% for US, respectively. We have proposed a US-based predictive scoring model for RT-treated cervical LN evaluations [15]. The formula is 1.35 × (long axis) + 1.48 × (echogenic hilum) + 2.03 × (short axis) + 2.27 × (margin) + 3.7. The cut-off value is set at 7, with lymphadenopathy classified as malignant if the score is higher than or equal to 7. The sensitivity is 86%, specificity is 80%, and accuracy is 83%. Previous studies have only focused on the accuracy of a single diagnostic modality. In this study, we aim to evaluate whether the addition of US +/− FNA to MRI/CT improves the diagnostic accuracy in evaluating cervical lymphadenopathy in irradiation-treated oral cancer patients.

## 2. Materials and Methods

The hospital’s Research Ethics Committee (No.109169-E) has approved the current study. The need for informed consent was waived by the Research Ethics Committee due to the retrospective and anonymous study design. The study was conducted in accordance with the World Medical Association Declaration of Helsinki and the Strengthening the Reporting of Observational Studies in Epidemiology (STROBE) statement and did not affect the treatment or outcome of the study group. All data are included in the supplementary information (Supplementary Tables S1–S3).

We retrospectively reviewed the data from oral cancers patients who received RT or CRT from February 2008 to November 2019 at a tertiary medical center. Oral cancer here refers only to cancers of the oral cavity and does not include oropharyngeal cancer. The study included patients who had lymphadenopathy, defined as the occurrence of cervical LN(s), either detected through palpation or imaging examination. We excluded patients without curative treatment before the US examination, without matching imaging of CT or MRI before or after the US study, or loss to follow-up. The B-mode and Doppler US were performed without contrast, and the size of the neck LNs, including short and long axis, as well as ratio of short-to-long axis (S/L), were recorded from B-mode US images. US-guided FNA was achieved in patients who had abnormal US features during the examination [3,15,16,17]. Finally, we included and analyzed the data of 104 patients with 104 LNs (Supplementary Table S1). The final diagnoses of recurrent LNs were depended on the pathological reports from a salvage operation, open biopsy, or core needle biopsy. A LN without FNA or with a negative cytopathological diagnosis was monitored for at least 12 months to confirm that there was no malignancy. During this surveillance period, diagnostic examinations, including MRI/CT and US with/without FNA, were performed every 6 months or when there was clinical enlargement of the LN.

The diagnostic examinations used in this study included MRI/CT, a post-RT US prediction model, and US + FNA. The MRI/CT was interpreted by experienced radiologists, while the US was performed and interpreted by experienced otolaryngologists. An enlarged short axis (≥10 mm), irregular margin, or central necrosis of a LN was considered to be malignant under MRI/CT [18]. If the score of the post-RT US predictive model was ≥7, the node was regarded as malignant [15]. The result of the US + FNA was defined by the cytopathological report and the post-RT US predictive model. If the FNA was not performed, the result was based solely on the post-RT US predictive model. In this study, we assessed these three modalities: (1) MRI/CT, (2) MRI/CT with a post-RT US predictive model, and (3) MRI/CT with US + FNA (Supplementary Table S2).

The continuous variable was expressed as the mean ± standard deviation (SD) and compared using the two-sample *t*-test, while the categorical variable was expressed as the number (percent) and analyzed using either a χ^2^ test or Fisher’s exact test. The odds ratio (OR) with a 95% confidence interval (CI) was reported. The predicted probability of the recurrent LN for different diagnostic methods was calculated using logistic regression analysis (Supplementary Table S3). The performances of the different diagnostic methods were assessed by comparing the receiver operating characteristic (ROC) curve and the area under the ROC curve (C-statistics). *p*-vales < 0.05 were interpreted as being statistically significant different. All statistical analyses were compared by using SPSS Statistics for Windows, Version 28.0. (IBM Corp., Armonk, NY, USA).

## 3. Results

In total, 104 patients with RT-treated oral cancer who had neck lymphadenopathy were recruited (Table 1). Of the 104 patients, 94 (90%) were male patients, and 10 (10%) were female. Their ages ranged from 29 to 91 years, with a mean age of 55 ± 11 years. The mean duration between the completion of RT and the imaging examination was 504 ± 704 days. Based on the definite diagnoses, there were 68 (65%) non-recurrent patients and 36 (35%) recurrent patients. The most common primary cancer subsites were the tongue, buccal area, and gingiva, and the most lymphadenopathies were located at levels I, II, and III.

The basic demographic and clinical data of these recurrent and non-recurrent patients are compared in Table 2. Significant differences were found between patients with recurrent and non-recurrent lymphadenopathy in the duration between RT/CRT and the imaging examination (*p* = 0.001), long axis (*p* < 0.001), short axis (*p* < 0.001), S/L (*p* = 0.003), margin (*p* < 0.001), calcification (*p* = 0.022), internal echogenicity (*p* < 0.001), echogenic hilum (*p* < 0.001), and vascular pattern (*p* = 0.013). However, no significant differences were observed in age, sex, primary cancer subsite, level of lymphadenopathy, echogenicity, and architecture.

Table 3 shows a comparison of the diagnostic examinations used to evaluate neck lymphadenopathy in oral cancer patients who have undergone radiation therapy. There were significant differences in differentiating between malignant (recurrent) nodal disease and benign (non-recurrent) cervical LN in all three diagnostic examinations (all *p*-values < 0.001). In assessing recurrent LN in oral cancer patients after neck irradiation, the highest OR was found in the US + FNA group (415.4). The MRI/CT group had an OR of 37.8, while the post-RT US predictive model had an OR of 23.9.

Table 4 presents the predicted probability of malignancy for MRI/CT, MRI/CT with a post-RT US predictive model, and MRI/CT with US + FNA. If one patient had a MRI/CT examination showing LN recurrence, the predicted probability of malignancy was 89.3%. If both MRI/CT and the post-RT US predictive model results were malignancy, the predicted probability of malignancy could increase to 96.0%. If someone had MRI/CT and US + FNA results, both presenting a recurrent LN, the predicted probability of malignancy would be 100%.

**Figure 1:**
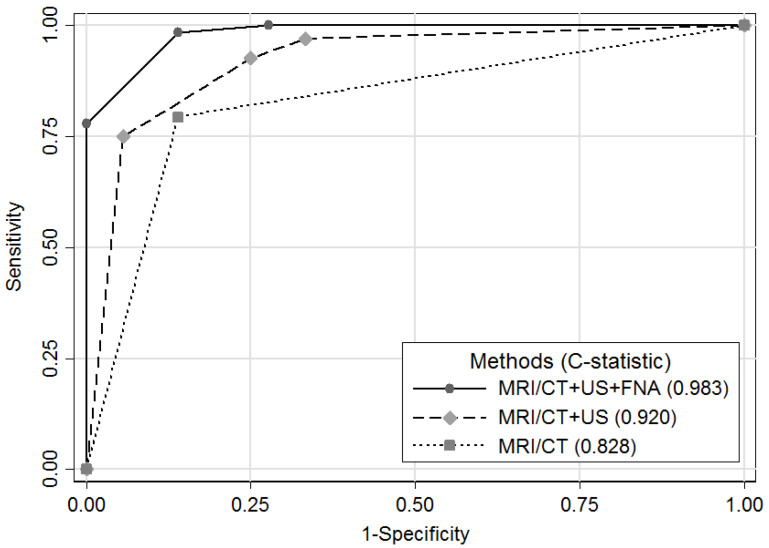
Receiver operating characteristic (ROC) curves of MRI/CT, MRI/CT with a post-RT US predictive model, and MRI/CT with US + FNA.

As illustrated in Figure 1, when we compared the diagnostic performance between these examinations by ROC curve, we found that the areas under the ROC curve (C-statistic, 95% CI) were highest in MRI/CT with US + FNA (0.983, 0.967~0.999), followed by MRI/CT with a post-RT US predictive model (0.920, 0.864~0.976), and then MRI/CT (0.828, 0.753~0.903).

## 4. Discussion

Imaging examinations are an important tool during the surveillance of oral cancer patients. Several studies have pointed out that the timely diagnosis of recurrent lymphadenopathy can lead to early salvage treatment, such as surgery or RT, which can improve survival outcomes [7,19,20]. Nevertheless, physicians may have difficulty in evaluating an enlarged node due to tissue fibrosis or scarring after neck irradiation [2,4]. Only some research has compared the performance of US and MRI/CT in detecting nodal recurrence during post-treatment surveillance in patients with head and neck cancers [13,14]. As far as we know, there is no previous study that investigated the diagnostic effect of the addition of a US examination to the currently used MRI/CT examination during follow-up in RT-treated oral cancer. As demonstrated from Figure 1, combining MRI/CT with either US + FNA or a post-RT US predictive model results in higher C-statics than only using MRI/CT (0.98 or 0.92 vs. 0.83), representing that the addition of a US examination to a MRI and/or CT is beneficial for the detection of recurrent LN in patients with irradiation-treated oral cancers. Furthermore, in Table 4, we demonstrated that the predicted malignancy rate was 7.3% if the diagnostic results of the MRI/CT and the post-RT US predictive model were both negative for malignancy, and this rate further decreased to 0% if the diagnostic results of the MRI/CT and US + FNA were both negative. On the contrary, the predicted malignancy rate was 31.1% if the diagnostic result of the MRI/CT alone was negative for malignancy.

Imaging examinations for surveillance were first performed around 3 months after a definitive treatment and then annually, as suggested by the NCCN guidelines. In our hospital, we performed a MRI/CT and US during these time points. If patients were found to have lymphadenopathy, either through palpation or imaging examination, we shortened the interval of the imaging examinations to every 6 months or when there was clinical enlargement of the LN. Our study found that regularly adding a US to MRI/CT is beneficial for the early diagnosis of nodal malignancy. However, further studies are needed to determine the most appropriate time interval for imaging examinations.

To better increase the diagnostic accuracy of US for evaluating the nodal status and to overcome the diagnostic heterogeneity among different physicians, we proposed a US predictive model for RT-treated cervical LN evaluations in a previous study [15]. The model was as follows: 1.35 × (long axis) + 1.48 × (echogenic hilum) + 2.03 × (short axis) + 2.27 × (margin) + 3.7. In our previous study, the post-RT US predictive model attained a diagnostic performance of 86% sensitivity, 80% specificity, and 83% accuracy. In this study, the model achieved 92.7% sensitivity, 75.0% specificity, and 86.5% overall accuracy. Although we tried to minimize the factor of operator dependence, there were still subjective features, such as the margin and echogenic hilum, that needed to be interpreted by physicians, which might inevitably cause a diagnostic disagreement. However, our study still provided a structured method for diagnosing the nodal status using US in oral cancer patients after radiotherapy, with an acceptable diagnostic performance.

US-guided FNA is conducted when there are suspicions of recurrent cervical lymphadenopathy during the US examination. The US features of suspicious recurrent cervical lymphadenopathy include large size, round shape, irregular boundary, calcification, cystic change, central necrosis, heterogeneous internal echogenicity, absent echogenic hilum, and a peripheral or mixed vascular pattern [3,15,16,21], and our study also reported significant differences in some of these parameters between recurrent and non-recurrent patients (Table 2). In this study, the most accurate way to determine a recurrent LN in irradiation-treated oral cancer was utilizing the diagnostic examinations of the combination of MRI/CT with US + FNA. In clinical practice, clinicians frequently prefer to observe the serial changes of a physical examination and imaging modalities, including MRI, CT, and PET, to evaluate the neck after RT. However, without additional tissue confirmation, this watchful waiting approach can occasionally cause the risk of a delayed diagnosis and treatment. Our results further elucidate that, during the evaluation of neck LN in irradiation-treated oral cancer patients, the false negative rate is 20.6% (14/68) in those who are diagnosed using MRI/CT alone (Table 3) and 0% (0/68) in those who are assessed using MRI/CT combined with US + FNA. Therefore, we recommend obtaining FNA concurrently during a US examination when there are suspicions of ultrasonographic characteristics of a LN in patients with irradiation-treated oral cancer.

**Figure 2:**
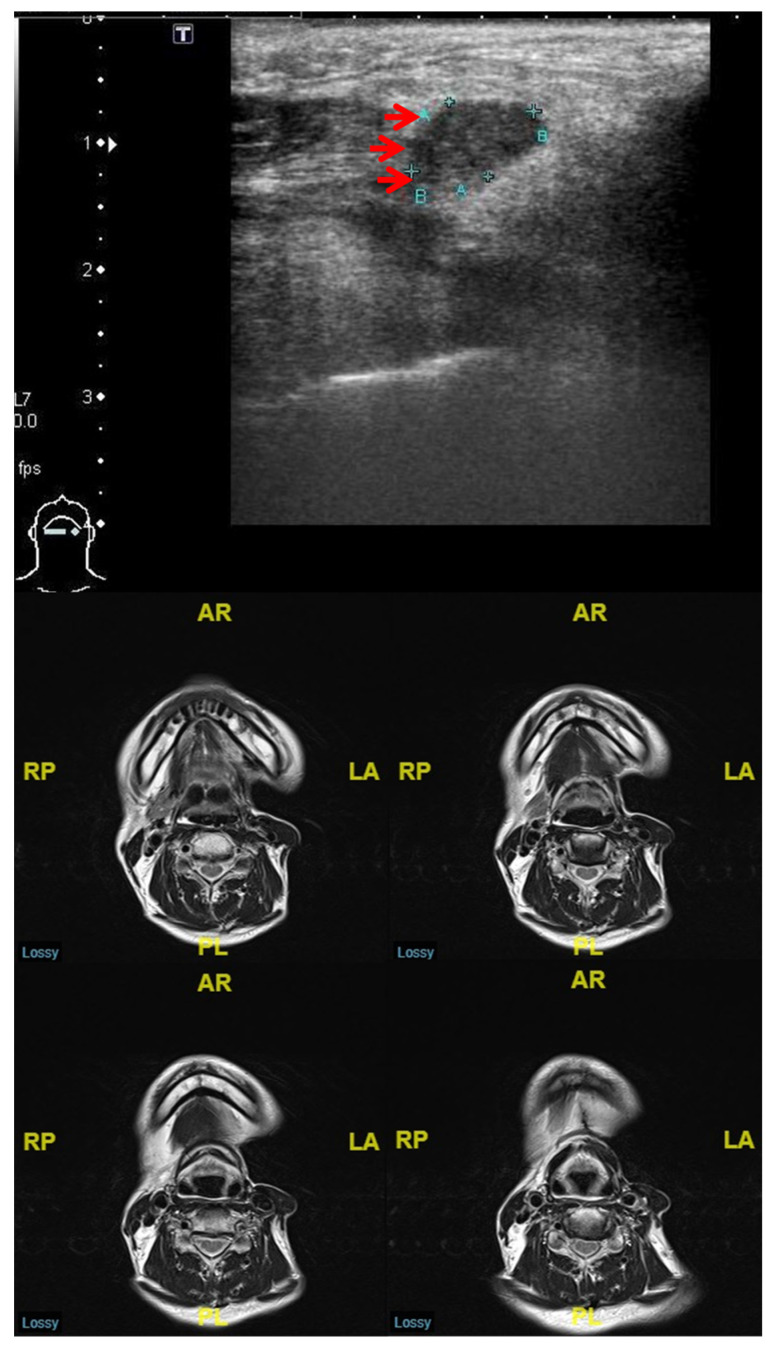
This is a 51-year-old male who was diagnosed with left tongue cancer status post-wide excision, left neck dissection, and adjuvant CRT. The 5-month post-treatment MRI reported no abnormal cervical LN (lower part of image). The US examination revealed a hypoechogenic mass (0.66 × 1.07 cm) over the right neck level IA (arrow, upper part of image). Both the cytological and pathological results demonstrated a metastatic LN.

On the other hand, our study also shows that the NPV of MRI/CT (68.9% (31 out of 45)) is lower than that of the post-RT US predictive model (84.4% (27 out of 32)) (Table 3). We believe that the treatment-related effects may have caused this outcome. First, the LNs detected after neck radiation have a tendency to be more heterogeneous with a lower radiopacity in CT imaging with contrast enhancement [21]. Second, we have demonstrated that the size of a malignant LN is smaller in irradiated compared to in non-irradiated oral cancer patients [17]. Using a 3-mm slice CT or 5-mm slice MRI, it is sometimes not easy to detect a small lymphadenopathy. Accordingly, after the CT or MRI examination, smaller and minor enhanced recurrent lymphadenopathy might be categorized as benign, resulting in a lower NPV (Figure 2). On the other hand, US is a real-time examination with high-resolution continuous images, and it can be used to evaluate a small LN thoroughly with the assistance of the US-based prediction model [15,17,22].

PET/CT is another important imaging tool for the post-treatment surveillance of oral cancer patients. The NCCN guidelines suggest performing a PET/CT within 3–6 months after oral cancer treatment and then annually. However, a PET/CT is not routinely performed in our country, because it is not covered by health insurance for just surveillance. For patients with head and neck cancer after radiotherapy, the sensitivity and specificity of a PET/CT for detecting nodal malignancy are 72.3% and 88.3%, respectively [23]. The principle of a PET/CT is based on the physical uptake of FDG. However, some inflammations may be mistaken for malignancy, causing false positives. Additionally, if there is no or a reduced FDG uptake at the tumor site, false negatives may also occur [24]. In such situations, a US is helpful in checking the nodal status [25,26].

Our study had several limitations. First, the study was a retrospective design, which might include unnoticed selection biases and unmeasured confounding factors. Second, definite diagnoses of non-recurrent LNs were not all obtained through histopathological results. Some LNs with negative cytopathological diagnoses and negative MRI/CT results were followed for at least 12 months to make sure that no recurrence had occurred. Third, whether or not to execute a FNA was determined by physicians based on suspicious US features and their experiences. Therefore, the most appropriate timing to perform a FNA could not be obtained here. This study’s strength was that it was a single-center cohort of homogeneous patients and treatment (oral cancer patients after complete treatment, including RT or CRT), which enabled an in-depth evaluation and comprehensive data review of all patients.

## 5. Conclusions

In conclusion, we suggest that a US examination is a beneficial tool to detect nodal recurrence in patients with irradiation-treated oral cancers. Either a post-RT US predictive model or US + FNA combined with MRI/CT can increase the diagnostic performance of a recurrent LN in this patient group.

## Figures and Tables

**Table 1:** Demographic data of the included patients and clinical data of their lymphadenopathy.

Parameters	*n* (%) or Mean ± SD
Age (years)	55 ± 11
Sex (Female/Male)	10 (10%)/94 (90%)
Duration between RT/CRT and imaging examination (days)	504 ± 704
Examinations that patients received	
MRI/CT	4 (4%)/100 (96%)
US	104 (100%)
FNA	93 (89%)
Primary cancer subsite	
Tongue/Buccal area/Gingiva/ Palate/Mouth floor/Retromolar trigone	51 (49%)/28 (27%)/13 (12%)/5 (5%)/1 (1%)/4 (4%)
Level of lymphadenopathy	
I/II/III	43 (41%)/26 (25%)/14 (13%)
IV/V/VI	6 (6%)/8 (8%)/7 (7%)
Sonographic feature of lymphadenopathy	
Short axis (cm)	1.07 ± 0.57
Long axis (cm)	1.66 ± 0.89
S/L	0.66 ± 0.16
Margin (Clear/Vague)	58 (56%)/46 (44%)
Internal echogenicity (Homo-/Hetero-geneous)	47 (45%)/57 (55%)
Echogenicity (Hyper- or Iso-/Hypo-echogenicity)	5 (5%)/99 (95%)
Calcification (Absent/Present)	95 (91%)/9 (9%)
Architecture (Cystic/Solid)	20 (19%)/84 (81%)
Echogenic hilum (Absent/Present)	85 (82%)/19 (18%)
Vascular pattern (Hilar or Avascular/Others)	88 (85%)/15 (15%)

Abbreviations: RT/CRT, radiotherapy/chemoradiation therapy; MRI, magnetic resonance imaging; CT, computed tomography; US, ultrasound; FNA, fine needle aspiration; S/L, ratio of short-to-long axis.

**Table 2:** Comparison of the demographic and clinical data between recurrent and non-recurrent oral cancer patients.

Parameters, *n* (%) or Mean ± SD	Recurrent Patients (*n* = 68)	Non-Recurrent Patients (*n* = 36)	*p*-Value
Age (years)	57 ± 12	53 ± 9	0.084
Sex			0.398
Female	8 (12%)	2 (6%)	
Male	60 (88%)	34 (94%)	
Duration between RT/CRT and imaging examination (days)	344 ± 486	806 ± 929	0.001 *
Examination that patients received			0.680
MRI	65 (96%)	35 (97%)	
CT	3 (4%)	1 (3%)	
Primary cancer subsite			0.393
Tongue	33 (49%)	18 (50%)	
Buccal area	15 (22%)	13 (36%)	
Gingiva	10 (15%)	3 (8%)	
Palate	3 (4%)	2 (6%)	
Lip	1 (1%)	0 (0%)	
Mouth floor	4 (6%)	0 (0%)	
Retromolar trigone	2 (3%)	0 (0%)	
Level of lymphadenopathy			0.290
I	23 (33%)	20 (55%)	
II	21 (31%)	5 (14%)	
III	10 (15%)	4 (11%)	
IV	4 (6%)	2 (6%)	
V	6 (9%)	2 (6%)	
VI	4 (6%)	3 (8%)	
Sonographic feature of lymphadenopathy			
Short axis (cm)	1.28 ± 0.56	0.67 ± 0.33	<0.001 *
Long axis (cm)	1.92 ± 0.93	1.17 ± 0.57	<0.001 *
S/L	0.69 ± 0.15	0.60 ± 0.18	0.003 *
Margin			<0.001 *
Clear	27 (40%)	31 (86%)	
Vague	41 (60%)	5 (14%)	
Internal echogenicity			<0.001 *
Homogenous	16 (24%)	31 (86%)	
Heterogeneous	52 (76%)	5 (14%)	
Echogenicity			0.795
Hyper or Iso	3 (4%)	2 (6%)	
Hypo	65 (96%)	34 (94%)	
Calcification			0.022 *
Absent	59 (87%)	36 (100%)	
Present	9 (13%)	0 (0%)	
Architecture			0.126
Cystic	16 (24%)	4 (11%)	
Solid	52 (76%)	32 (89%)	
Echogenic hilum			<0.001 *
Absent	64 (94%)	21 (58%)	
Present	4 (6%)	15 (42%)	
Vascular pattern			0.013 *
Hilar or avascular	53 (79%)	35 (97%)	
Other	14 (21%)	1 (3%)	

Abbreviations: RT/CRT, radiotherapy/chemoradiation therapy; MRI, magnetic resonance imaging; CT, computed tomography; US, ultrasound; S/L, ratio of short-to-long axis. * Statistically significant.

**Table 3:** Comparison of the sensitivity, specificity, accuracy, and odds ratio among single diagnostic examinations in the evaluation of neck lymphadenopathy in post-irradiation oral cancer patients.

Diagnostic Examinations	Definite Diagnosis			Sensitivity (95% CI)	Specificity (95% CI)	Accuracy (95% CI)	OR (95% CI)
	Malignancy	Benignity	*p*-Value				
MRI/CT result				79.4% (69.8–89.0%)	86.1% (74.8–97.4%)	81.7% (74.3–89.2%)	23.9 (7.9–72.7)
Malignancy	54 (79%)	5 (14%)	<0.001 *				
Benignity	14 (21%)	31 (86%)					
Post-RT US predictive model ^†^ result				92.7% (86.4–98.9%)	75.0% (60.9–89.1%)	86.5% (80.0–93.1%)	37.8 (11.6–123.3)
Malignancy (score ≥ 7)	63 (93%)	9 (25%)	<0.001 *				
Benignity (score < 7)	5 (7%)	27 (75%)					
US + FNA result				98.5% (95.7–100%)	86.1% (74.8–97.4%)	94.2% (89.7–98.7%)	415.4 (46.5–3707.4)
Malignancy	67 (99%)	5 (14%)	<0.001 *				
Benignity	1 (1%)	31 (86%)					

Abbreviations: MRI, magnetic resonance imaging; CT, computed tomography; RT, radiotherapy; US, ultrasound; FNA, fine needle aspiration. ^†^ The formula is 1.35 × (long axis) + 1.48 × (echogenic hilum) + 2.03 × (short axis) + 2.27 × (margin) + 3.7. * Statistically significant.

**Table 4:** The predicted probability of the recurrent LN among MRI/CT, MRI/CT with a post-RT US predictive model, and MRI/CT with US + FNA by using logistic regression analysis.

Diagnostic Modalities	Diagnostic Result of Each Examination			Predicted Probability of Malignancy
	MRI/CT	Post-RT US Predictive Model	US + FNA	
MRI/CT	Malignancy			91.5%
	Benignity			31.1%
MRI/CT with a post-RT US predictive model	Malignancy	Malignancy		96.0%
	Benignity	Malignancy		63.7%
	Malignancy	Benignity		51.8%
	Benignity	Benignity		7.3%
MRI/CT with US + FNA	Malignancy		Malignancy	100%
	Benignity		Malignancy	73.7%
	Malignancy		Benignity	16.7%
	Benignity		Benignity	0%

Abbreviations: MRI, magnetic resonance imaging; CT, computed tomography; RT, radiotherapy; US, ultrasound; FNA, fine needle aspiration.

## Data Availability

The data presented in this study are available in the Supplementary Materials.

## Supplementary Materials

The following supporting information can be downloaded at: https://www.mdpi.com/article/10.3390/diagnostics13142409/s1, Table S1: The data of Table 1 and Table 2; Table S2: The data of Table 3; Table S3: The data of Table 4.
